# Estimated Failure to Report Unsuccessful Quit Attempts by Type of Cessation Aid: A Population Survey of Smokers in England

**DOI:** 10.1155/2022/5572480

**Published:** 2022-04-09

**Authors:** Olga Perski, Robert West, Jamie Brown

**Affiliations:** Department of Behavioural Science and Health, University College London, 1-19 Torrington Place, London WC1E 6BT, UK

## Abstract

**Introduction:**

It has been estimated that smokers tend to fail to report unsuccessful quit attempts that lasted a short time and occurred a longer time ago. However, it is unclear whether the failure to report unsuccessful quit attempts varies by the type of cessation aid used.

**Methods:**

A total of 5,892 smokers aged 16+ years who had made 1+ quit attempts in the past year were surveyed between January 2014 and December 2020 as part of the Smoking Toolkit Study. Respondents indicated when their most recent quit attempt started, how long it lasted, and which cessation aid(s) were used (e.g., unaided, varenicline, and behavioural support). The percentage failure to report for each cessation aid and 95% bootstrap confidence intervals (CIs) were estimated with an established method. Test for equality of proportions was performed to examine whether quit attempts lasting between one day and one week and that started >6 months ago failed to be reported at a different rate depending on the cessation aid used.

**Results:**

We estimated that after three months, 97% (95% CI = 96%-98%) of unaided quit attempts lasting less than one day, 80% (95% CI = 79%-81%) of those lasting between one day and one week, and 60% (95% CI = 59%-61%) of those lasting between one week and one month fail to be reported. Compared with unaided attempts, the estimated percentage failure to report quit attempts that lasted between one day and one week and that started >6 months ago was significantly lower for attempts involving behavioural support (92% of unaided attempts vs. 75% of attempts involving behavioural support, *χ*^2^(1) = 9.29, *p* = 0.002). No other significant differences were detected.

**Conclusions:**

Smokers in England appear to fail to report a substantial proportion of unsuccessful quit attempts. This failure appears particularly prominent for attempts that last a short time or occurred longer ago and appears lower for attempts involving behavioural support compared with unaided attempts.

## 1. Introduction

Survey data can measure key aspects of the smoking cessation process, including the frequency and duration of quit attempts and the popularity and relative effectiveness of available cessation aids. For example, population surveys indicate that ~40% of smokers report having made at least one quit attempt in any given year [1]. The majority of quit attempts are unaided [2]; however, smokers who report using any type of cessation aid are more frequently using pharmacological support or e-cigarettes compared with, for example, face-to-face behavioural support or digital aids [3, 4]. The utility of survey data largely depends on the accuracy of respondents' quit attempt histories. However, a nontrivial level of misreporting of quit attempt histories likely occurs. Such misreporting is particularly relevant for unsuccessful quit attempts (as people tend to remember successful attempts, e.g., through identifying as ex-smokers), which can have severe consequences for the estimation of the effectiveness of different quit aids and tobacco control policies [5].

Memory processes such as forgetting are relevant to the misreporting of unsuccessful quit attempts in general and unsuccessful attempts involving specific cessation aids in particular [6]. First, forgetting may simply occur due to decreasing accessibility of stored information over time. For example, Berg and colleagues used data from the English Smoking Toolkit Study to assess whether smokers' estimated failure to report quit attempts varied as a function of the duration of the quit attempt and time since the quit attempt started. They estimated a strong trend for quit attempts that lasted for shorter periods and that started a longer time ago to fail to be reported [7]. Second, inattentive or shallow processing of information may lead to the weak encoding of memories. A recent study in England found that reports on the use of digital aids in a smoking cessation attempt was low at 2.7% [4]. Smokers may fail to report unsuccessful attempts involving digital aids at a higher rate than attempts involving pharmacological support or contact with a healthcare professional, as the use of digital aids is typically discontinued during the first week of download [8]. Moreover, better recall of failed quit attempts among smokers using stop smoking medications compared with self-quitters has been observed [5], which can lead to the underestimation of the effectiveness of certain quit aids. Third, current beliefs and retrospective distortions may influence the encoding of memories. For example, the belief that a cessation aid was not personally relevant or useful for quitting may lead to forgetting [9]. Fourth, smokers may also reinterpret quit attempts that failed more quickly as not being “real attempts” [7].

Using data from the English Smoking Toolkit Study, we aimed to extend previous findings by examining whether the estimated failure to report unsuccessful quit attempts varies by the type of cessation aid used.

## 2. Methods

### 2.1. Study Design and Setting

The study protocol and analysis plan were preregistered on the Open Science Framework (osf.io/k6q3d). This was a correlational study involving cross-sectional survey data. The STROBE guidelines were used in the design and reporting of the study [10]. The study is part of the ongoing Smoking Toolkit Study (STS), which involves monthly, face-to-face, computer-assisted household surveys with adults aged 16+ in England [11]. The STS uses a hybrid of random probability and quota sampling, which results in a sample that is representative of the adult population of smokers in England. Interviews are held with one household member. Informed consent is obtained prior to each interview. Ethical approval was granted by UCL's Research Ethics Committee (2808/005).

### 2.2. Study Population

Data were collected from respondents surveyed between January 2014 (the first wave at which the use of e-cigarettes had stabilised—selected as starting point to reduce potential bias introduced by the increased popularity of e-cigarettes) and December 2020 (the latest wave of data available at the time of analysis). Respondents were included in the analyses if they were aged 16+ years, a current smoker, had made at least one serious quit attempt in the past year, and had complete data on the demographic and smoking variables of interest. This deviated from the preregistered study protocol, in which we had specified that recent ex-smokers would also be included. However, by definition, they cannot have forgotten their most recent quit attempt.

### 2.3. Measures

Respondents were asked to provide data on sex; age; social grade, measured with the British National Readership Survey's Social Grade Classification Tool [12]; cigarettes per day and time to first cigarette [13]; and the number of serious quit attempts made in the past year (defined as deciding to try to never smoke again).

Smokers who made at least one serious quit attempt in the past year were asked to select which of the following cessation aids were used in their most recent quit attempt: (1) prescription nicotine replacement therapy (NRT), (2) NRT bought over the counter (OTC), (3) varenicline, (4) bupropion, (5) e-cigarettes, (6) face-to-face behavioural support, (7) telephone support, (8) written self-help materials, (9) digital support (i.e., websites/smartphone apps), (10) hypnotherapy, and (11) none of the above (“unaided”). Respondents were asked to indicate when their most recent quit attempt started, with response options including: (1) 1-7 days ago, (2) 8-30 days ago, (3) 31-60 days ago, (4) 61-90 days ago, (5) 91 days to 6 months ago, (6) >6 months to 1 year ago, and (7) don't know/not stated. Those reporting “don't know/not stated” were excluded. Finally, respondents were asked how long their most recent quit attempt lasted, with response options including (1) still not smoking, (2) <1 day, (3) 1-7 days, (4) 8-30 days, (5) 31-60 days, (6) 61-90 days, (7) 91 days to 6 months, (8) >6 months to 1 year, and (9) don't know/not stated. Those reporting “still not smoking” or “don't know/not stated” were excluded. Responses that fell outside the realms of possibility (e.g., respondents indicating that their quit attempt started 1-7 days ago and lasted >6 months to 1 year) were also excluded. This had not been specified in the preregistered analysis plan.

### 2.4. Data Analysis

Data were analysed in RStudio v.1.2.5. The percentage “failure to report” was estimated for each cessation aid with an established analytic approach [7]. We first standardised each of the temporal assessment periods to reflect the number of quit attempts at the population-level (and not per individual) that would be reported if all periods were one month long. For example, the number of quit attempts that started in the last week was multiplied by 4 to reflect the number of quit attempts that would be expected to occur over a 1-month period at the same rate. Longer time periods were divided appropriately.

The estimated “failure to report” was then derived by calculating the percentage of quit attempts of different lengths that failed to be reported for each time period, assuming that the rate of reporting for attempts that started most recently (e.g., in the last week) is most accurate and that the rate of quit attempts over time is uniform. For example, the percentage failure to report for quit attempts that started 8-30 days ago and lasted for 1-7 days was derived by dividing the standardised number of quit attempts by the number of quit attempts that started in the last week and lasted for 1-7 days.

Matrices of time since the quit attempts started by length of the quit attempts with percentages estimated failure to report and 95% bootstrap percentile confidence intervals (CIs) were produced and plotted for each cessation aid. We performed 1000 bootstrap replications, with the 2.5th and 97.5th percentiles of the empirical distribution forming the 95% bootstrap percentile CIs [14]. Plots were visually inspected to examine whether the estimated forgetting curves were differentially shaped for any of the cessation aids. In addition, tests for equality of proportions were performed with the *prop.test* function to examine whether quit attempts lasting between one day and one week and that started >6 months ago failed to be reported at a different rate depending on the cessation aid used. As previous research had suggested that smokers tend to fail to report unsuccessful quit attempts that lasted a short time and occurred a longer time ago [7], we reasoned that if there is any moderation by cessation aid, it would be important to detect this for attempts that tend to be forgotten at a high (rather than low) rate. This had not been specified in the preregistered analysis plan.

As relapse curves differ by cessation aid (i.e., some aids are more effective) [15] [16–18], we considered applying an “effectiveness adjustment” to the raw quit attempt numbers using estimates from [15, 16]. However, as the effectiveness of each aid is already considered in the original analytic approach (with the ratio of expected vs. reported quit attempts estimated on the basis of smokers whose quit attempts lasted the same amount of time and “conferred” the same level of effectiveness at that moment), the effectiveness adjustment did not alter the percentages (see Supplementary File 1).

#### 2.4.1. Planned Sensitivity Analyses

As there was an increasing trend in unaided quit attempts in England at the end of 2017 (http://www.smokinginengland.info/), we conducted a planned sensitivity analysis (SA) for unaided quit attempts using data from 2014 to the end of 2017 (see Supplementary File 2).

#### 2.4.2. Unplanned Sensitivity Analyses

We also conducted two unplanned SAs, examining the percentage estimated failure to report when combining all respondents who used any pharmacological aid or any behavioural aid (excluding those who used both a pharmacological and a behavioural aid; “multiple aids”) to increase sample sizes (see Supplementary File 2).

## 3. Results

A total of 6,614 smokers who had made at least one serious quit attempt in the past year were surveyed, of whom 70 respondents had missing data on any of the demographic or smoking characteristics of interest, with a further 652 respondents with out of range or implausible combinations of started/lasted values, yielding a total sample of 5,892 (89.1%) respondents with complete data on all variables of interest. The majority of unsuccessful quit attempts lasted less than one month (see Table 1).

**Figure 1:**
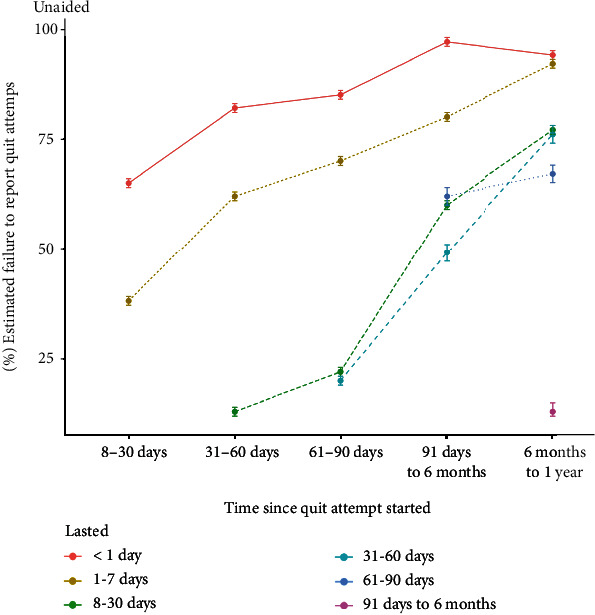
Percentage estimated failure to report quit attempts of varying lengths (indicated by the line colour) and varying times since the quit attempt started (*x*-axis).

The matrix of time since the quit attempt started by length of the quit attempt and percentages estimated failure to report for unaided attempts is presented in Table 2 and Figure 1. Supplementary File 2 illustrates the rate of estimated failure to report for each cessation aid. We were unable to estimate the rate of failure to report for attempts involving bupropion, telephone support, written support, and hypnotherapy due to small sample sizes (i.e., >3 cells with 0 reported quit attempts). In addition, bootstrap CIs could not be estimated for percentages equal to 0 or 100. Overall, we estimate that a substantial proportion of unsuccessful quit attempts fail to be reported. This failure is particularly prominent for attempts that last a short time or occurred longer ago. Tests for equality of proportions indicated that, compared with unaided attempts, the percentage failure to report quit attempts that lasted between one day and one week and that started >6 months ago was significantly lower for attempts involving behavioural support (92% vs. 75%, *χ*^2^(1) = 9.29, *p* = 0.002). No other significant differences were detected (all *p*'s > 0.05; see Table 3).

## 4. Discussion

In smokers in England, we estimate that a substantial proportion of unsuccessful quit attempts fail to be reported. This failure appears particularly prominent for attempts that last a short time or occurred a long time ago. Compared with unaided attempts, the estimated percentage failure to report quit attempts that lasted between one day and one week and that started >6 months ago was significantly lower for attempts involving face-to-face behavioural support. Our results replicate those reported by Berg and colleagues a decade ago [7] and suggest that smokers may have somewhat poorer memory of unaided quit attempts compared with attempts involving behavioural support. A potential explanation for the estimated improved memory of unsuccessful attempts involving face-to-face behavioural support (compared with unaided attempts) may be due to such support involving a series of activities with emotional and cognitive salience (e.g., transportation to face-to-face meetings and conversations with and accountability to a healthcare professional).

Borland and colleagues have previously discussed how differential failure to report unsuccessful quit attempts may have consequences for the estimation of the effectiveness of treatments [5]. For example, we found that people appeared more likely to forget unsuccessful unaided attempts compared with those involving behavioural support. Insofar that this generalises, studies using retrospective surveys to estimate the comparative effectiveness of behavioural support will underestimate its effectiveness. Although clinical guidelines for smoking cessation are primarily underpinned by evidence from randomised controlled trials (which are not subject to differential failure to report), policy evaluations and related decisions sometimes rely on retrospective/cross-sectional survey data. This may lead to overestimations of policy effects, as the failure to report quit attempts that occurred a longer time ago (i.e., prior to the implementation of the new policy) contributes to the comparatively lower quit rates reported in the pre- compared with the postintervention period [5]. Therefore, the estimated failure to report quit attempts in the present study adds to the existing literature indicating that this is a serious issue for retrospective/cross-sectional survey data.

### 4.1. Limitations

First, respondents were only asked about “serious” quit attempts. However, it is plausible that a differently worded question may have captured a larger number of attempts, including those that (i) were “serious” at the outset but were retrospectively reclassified as less serious after having failed relatively quickly and (ii) were not regarded as “serious” by smokers at the outset but evolved into a sustained attempt to stop [19, 20]. Second, the popularity and effectiveness of different aids are likely to have impacted the results. We considered applying an “effectiveness adjustment” to the raw numbers but concluded that this would not alter the percentage estimated failure to report due to the analytic approach. Third, the results are dependent on the validity of the approach used to estimate the percentage failure to report quit attempts (i.e., the assumption that the rate of reporting for attempts that started in the last week is most accurate and that the rate of population-level quit attempts over time is uniform). For example, the estimate that 76% of those making an unaided quit attempt that lasted 2-3 months and that started 6-12 months ago would fail to report their attempt appears higher than expected and is substantially higher than the corresponding estimate (i.e., 8.5%) reported by Berg and colleagues [7]. It is likely that the estimates in the present study were sensitive to the small sample sizes for many of the quit aids, thus lacking precision, which limits strong conclusions. As it is difficult to test the validity of our method (i.e., there is no “gold standard” method for comparison), we recommend using triangulation across multiple methods and data sources to arrive at more precise forgetting estimates. However, it should be noted that the key assumption underpinning our method—i.e., that serious quit attempts that started in the last week should be accurately reported (which is grounded in decades of memory research)—supports its validity [6]. Fourth, as the cessation aids were not mutually exclusive (i.e., participants could indicate multiple options), this may have limited the ability to detect differences in the estimated percentage failure to report for the different cessation aids. Finally, our sample was young, the majority were light smokers, and due to low cell counts, we were unable to estimate the percentage failure to report for quit attempts involving bupropion, telephone support, written support, and hypnotherapy, which likely limits the generalisability of the results.

### 4.2. Implications and Future Directions

The overall finding that a large proportion of unsuccessful quit attempts may fail to be reported has implications for the assessment of quit attempt histories. Public health researchers should consider triangulating survey data with qualitative methods (e.g., following up smokers a period after their quit attempt) and ecological momentary assessments (i.e., brief, regular surveys delivered in or near real-time on people's mobile phones) [21], as this may help elucidate why smokers have poor memory of unsuccessful quit attempts. The finding that the estimated failure to report appeared lower for attempts involving face-to-face behavioural support compared with unaided attempts may be interpreted to suggest that interactions with stop smoking counsellors lead to deep information processing and hence strong encoding of memories [6]; however, this would need to be corroborated in future research.

## 5. Conclusion

In smokers in England, a substantial proportion of unsuccessful quit attempts may fail to be reported. This failure appears particularly prominent for attempts that last a short time or occurred longer ago and appears lower for attempts involving behavioural support compared with unaided attempts.

## Figures and Tables

**Table 1:** Participant demographic and smoking characteristics (*N* = 5,892).

Female, *n* (%)	2,943 (49.9%)
Age, *n* (%)	
16-24 years	1,204 (20.4%)
25-34 years	1,368 (23.2%)
35-44 years	1,038 (17.6%)
45-54 years	952 (16.2%)
55-64 years	744 (12.6%)
65+ years	586 (9.9%)
Social grade	
AB	792 (13.4%)
C1	1,706 (29.0%)
C2	1,337 (22.7%)
D	1,058 (18.0%)
E	999 (17.0%)
Time to first cigarette	
Do not know	36 (0.6%)
Within 5 minutes	932 (15.8%)
6-30 minutes	1,766 (30.0%)
31-60 minutes	1,211 (20.6%)
>60 minutes	1,947 (33.0%)
Cigarettes per day, mean (*SD*)	10.53 (7.74)
Time since unsuccessful quit attempt started, *n* (%)	
1-7 days ago	244 (4.1%)
8-30 days ago	598 (10.1%)
31-60 days ago	690 (11.7%)
61-90 days ago	799 (13.6%)
91 days to 6 months ago	1282 (21.8%)
> 6 months to 1 year ago	2279 (38.7%)
Length of unsuccessful quit attempt, *n* (%)	
< 1 day	529 (9.0%)
1-7 days	1312 (22.3%)
8-30 days	1846 (31.3%)
31-60 days	677 (11.5%)
61-90 days	521 (8.8%)
91 days to 6 months	478 (8.1%)
> 6 months to 1 year	529 (9.0%)
Cessation aid, *n* (%)^∗^	
Prescription NRT	254 (4.3%)
OTC NRT	1,125 (19.1%)
Varenicline	265 (4.5%)
Bupropion	39 (0.7%)
E-cigarettes	1,816 (30.8%)
Behavioural support	174 (3.0%)
Telephone support	32 (0.5%)
Written support	67 (1.1%)
Digital support	149 (2.5%)
Hypnotherapy	43 (0.7%)
Unaided	2,555 (43.4%)
Any pharmacological support (combined)	3,152 (53.5%)
Any behavioural support (combined)	407 (6.9%)
Multiple aids (i.e., any pharmacological and any behavioural support)	222 (3.8%)

NRT: nicotine replacement therapy; OTC: over the counter. ^∗^As cessation aids were not mutually exclusive (i.e., respondents could select multiple options), the total percentage exceeds 100%.

**Table 2:** Matrix of time since the unsuccessful quit attempt started by the length of the quit attempt and percentages estimated failure to report for unaided attempts (*n* = 2,555).

	Length of unsuccessful quit attempt						
	<1 day	1-7 days	8-30 days	31-60 days	61-90 days	91 days to 6 months	>6 months to 1 year
Time since unsuccessful quit attempt started							
1-7 days			—	—	—	—	—
Raw *n*	44	58					
Standardised *n*	176	232					
% failure to report	—	—					
8-30 days				—	—	—	—
Raw *n*	46	109	118				
Standardised *n*	61	145	157				
% failure to report	65%	38%	—				
31-60 days					—	—	—
Raw *n*	32	88	136	59			
Standardised *n*	32	88	136	59			
% failure to report	82%	62%	13%	—			
61-90 days						—	—
Raw *n*	27	69	123	47	52		
Standardised *n*	27	69	123	47	52		
% failure to report	85%	70%	22%	20%	—		
91 days to 6 months							—
Raw *n*	18	142	190	89	60	69	
Standardised *n*	6	47	63	30	20	23	
% failure to report	97%	80%	60%	49%	62%	—	
>6 months to 1 year							
Raw *n*	61	116	213	87	102	119	281
Standardised *n*	10	19	36	14	17	20	47
% failure to report	94%	92%	77%	76%	67%	13%	—

Raw *n*: the number of respondents indicating an unsuccessful quit attempt; Standardised *n*: the estimated number of unsuccessful quit attempts based on our calculations.

**Table 3:** Tests for equality of proportions for unsuccessful quit attempts that lasted between one day and one week and that started >6 months ago for the different quitting aids compared with unaided quit attempts.

Comparison	Percentage failure to report	*χ*2(1)	*p* value
Unaided vs. prescription NRT	92% vs. 83%	2.93	0.087
Unaided vs. OTC NRT	92% vs. 90%	0.06	0.805
Unaided vs. varenicline	92% vs. 83%	2.93	0.087
Unaided vs. e-cigarettes	92% vs. 88%	0.50	0.480
Unaided vs. behavioural support	92% vs. 75%	9.29	0.002
Unaided vs. digital support	92% vs. 83%	2.93	0.087

## Data Availability

The data underpinning the findings of this study are available on request from the senior author, JB.

## Supplementary Materials

Supplementary 1 Supplementary File 1: matrix of time since the quit attempt started by the length of the quit attempt and percentages estimated failure to report for unaided attempts, without and with an “effectiveness adjustment.”

Supplementary 2 Supplementary File 2: percentage estimated failure to report quit attempts of varying lengths (indicated by the line colour) and varying times since the quit attempt started (*x*-axis) for each cessation aid, any pharmacological aid, any behavioural aid, or unaided attempts with data up to 2017 (sensitivity analyses).
