# Supplementary material for: Estimated Failure to Report Unsuccessful Quit Attempts by Type of Cessation Aid: A Population Survey of Smokers in England
Source: J Smok Cessat. 2022 Apr 9;2022:5572480. doi: 10.1155/2022/5572480 (PMC9762728; doi:10.1155/2022/5572480)
Supplement: Supplementary 1 — Supplementary File 1: matrix of time since the quit attempt started by the length of the quit attempt and percentages estimated failure to report for unaided attempts, without and with an “effectiveness adjustment.” [file 5572480.f1.docx]

| **Supplementary File 1** |  |  |  |  |  |  |  |
| --- | --- | --- | --- | --- | --- | --- | --- |
|  |  |  |  |  |  |  |  |
| **Unaided** | **Length** |  |  |  |  |  |  |
| **When started** | 1) < 1 day | 2) 1-7 days | 3) 8-30 days | 4) 31-60 days | 5) 61-90 days | 6) 91 days to 6 months | 7) >6 months to 1 year |
| 1) 1-7 days |  |  | - | - | - | - | - |
| Raw n | 44 | 58 |  |  |  |  |  |
| Standardised n | 176 | 232 |  |  |  |  |  |
| % Failure to report | - | - |  |  |  |  |  |
| 2) 8-30 days |  |  |  | - | - | - | - |
| Raw n | 46 | 109 | 118 |  |  |  |  |
| Standardised n | 61,33 | 145 | 157 |  |  |  |  |
| % Failure to report | 65,2% | 37,4% | - |  |  |  |  |
| 3) 31-60 days |  |  |  |  | - | - | - |
| Raw n | 32 | 88 | 136 | 59 |  |  |  |
| Standardised n | 32 | 88 | 136 | 59 |  |  |  |
| % Failure to report | 81,8% | 62,1% | 13,6% | - |  |  |  |
| 4) 61-90 days |  |  |  |  |  | - | - |
| Raw n | 27 | 69 | 123 | 47 | 52 |  |  |
| Standardised n | 27 | 69 | 123 | 47 | 52 |  |  |
| % Failure to report | 84,7% | 70,3% | 21,8% | 20,3% | - |  |  |
| 5) 91 days to 6 months |  |  |  |  |  |  | - |
| Raw n | 18 | 142 | 190 | 89 | 60 | 69 |  |
| Standardised n | 6 | 47,33 | 63 | 30 | 20 | 23 |  |
| % Failure to report | 96,6% | 79,6% | 59,7% | 49,7% | 61,5% | - |  |
| 6) >6 months to 1 year |  |  |  |  |  |  |  |
| Raw n | 61 | 116 | 213 | 87 | 102 | 119 | 281 |
| Standardised n | 10 | 19 | 36 | 15 | 17 | 20 | 47 |
| % Failure to report | 94,2% | 91,7% | 77,4% | 75,4% | 67,3% | 13,8% | - |
|  |  |  |  |  |  |  |  |
|  |  |  |  |  |  |  |  |
|  |  |  |  |  |  |  |  |
|  |  |  |  |  |  |  |  |
| **Unaided - with effectiveness adjustment** | **Length** |  |  |  |  |  |  |
| **When started** | 1) < 1 day | 2) 1-7 days | 3) 8-30 days | 4) 31-60 days | 5) 61-90 days | 6) 91 days to 6 months | 7) >6 months to 1 year |
| 1) 1-7 days |  |  | - | - | - | - | - |
| Raw failed n | 44 | 58 |  |  |  |  |  |
| % continuous abstinent estimate for placebo from Jackson et al 2019 | 88,13 | 54,10 | 27,99 | 19,92 | 16,35 | 13,02 | 9,94 |
| Inflation to adjust raw failed for 'still not smoking' attempts | 49,93 | 107,22 |  |  |  |  |  |
| Standardised for time period n | 199,71 | 428,87 |  |  |  |  |  |
| % Failure to report | - | - |  |  |  |  |  |
| 2) 8-30 days |  |  |  | - | - | - | - |
| Raw n | 46 | 109 | 118 |  |  |  |  |
| % continuous abstinent estimate for placebo from Jackson et al 2019 | 88,13 | 54,10 | 27,99 | 19,92 | 16,35 | 13,02 | 9,94 |
| Inflation to adjust raw failed for 'still not smoking' attempts | 52,20 | 201,49 | 421,63 |  |  |  |  |
| Standardised for time period n | 69,59 | 268,66 | 562,18 |  |  |  |  |
| % Failure to report | 65,2% | 37,4% | - |  |  |  |  |
| 3) 31-60 days |  |  |  |  | - | - | - |
| Raw n | 32 | 88 | 136 | 59 |  |  |  |
| % continuous abstinent estimate for placebo from Jackson et al 2019 | 88,13 | 54,10 | 27,99 | 19,92 | 16,35 | 13,02 | 9,94 |
| Inflation to adjust raw failed for 'still not smoking' attempts | 36,31 | 162,67 | 485,95 | 296,25 |  |  |  |
| Standardised n | 36,31 | 162,67 | 485,95 | 296,25 |  |  |  |
| % Failure to report | 81,8% | 62,1% | 13,6% | - |  |  |  |
| 4) 61-90 days |  |  |  |  |  | - | - |
| Raw n | 27 | 69 | 123 | 47 | 52 |  |  |
| % continuous abstinent estimate for placebo from Jackson et al 2019 | 88,13 | 54,10 | 27,99 | 19,92 | 16,35 | 13,02 | 9,94 |
| Inflation to adjust raw failed for 'still not smoking' attempts | 30,64 | 127,55 | 439,50 | 236,00 | 318,05 |  |  |
| Standardised n | 30,64 | 127,55 | 439,50 | 236,00 | 318,05 |  |  |
| % Failure to report | 84,7% | 70,3% | 21,8% | 20,3% | - |  |  |
| 5) 91 days to 6 months |  |  |  |  |  |  | - |
| Raw n | 18 | 142 | 190 | 89 | 60 | 69 |  |
| % continuous abstinent estimate for placebo from Jackson et al 2019 | 88,13 | 54,10 | 27,99 | 19,92 | 16,35 | 13,02 | 9,94 |
| Inflation to adjust raw failed for 'still not smoking' attempts | 20,42 | 262,50 | 678,90 | 446,88 | 366,98 | 530,01 |  |
| Standardised n | 6,81 | 87,50 | 226,30 | 148,96 | 122,33 | 176,67 |  |
| % Failure to report | 96,6% | 79,6% | 59,7% | 49,7% | 61,5% | - |  |
| 6) >6 months to 1 year |  |  |  |  |  |  |  |
| Raw n | 61 | 116 | 213 | 87 | 102 | 119 | 281 |
| % continuous abstinent estimate for placebo from Jackson et al 2019 | 88,13 | 54,10 | 27,99 | 19,92 | 16,35 | 13,02 | 9,94 |
| Inflation to adjust raw failed for 'still not smoking' attempts | 69,22 | 214,43 | 761,09 | 436,84 | 623,86 | 914,08 | 2825,58 |
| Standardised n | 11,54 | 35,74 | 126,85 | 72,81 | 103,98 | 152,35 | 46,83 |
| % Failure to report | 94,2% | 91,7% | 77,4% | 75,4% | 67,3% | 13,8% | - |
