# Supplementary figures and images for: Estimated Failure to Report Unsuccessful Quit Attempts by Type of Cessation Aid: A Population Survey of Smokers in England
Source: J Smok Cessat. 2022 Apr 9;2022:5572480. doi: 10.1155/2022/5572480 (PMC9762728; doi:10.1155/2022/5572480)

**Supplementary File 2**

*Main analyses*

**
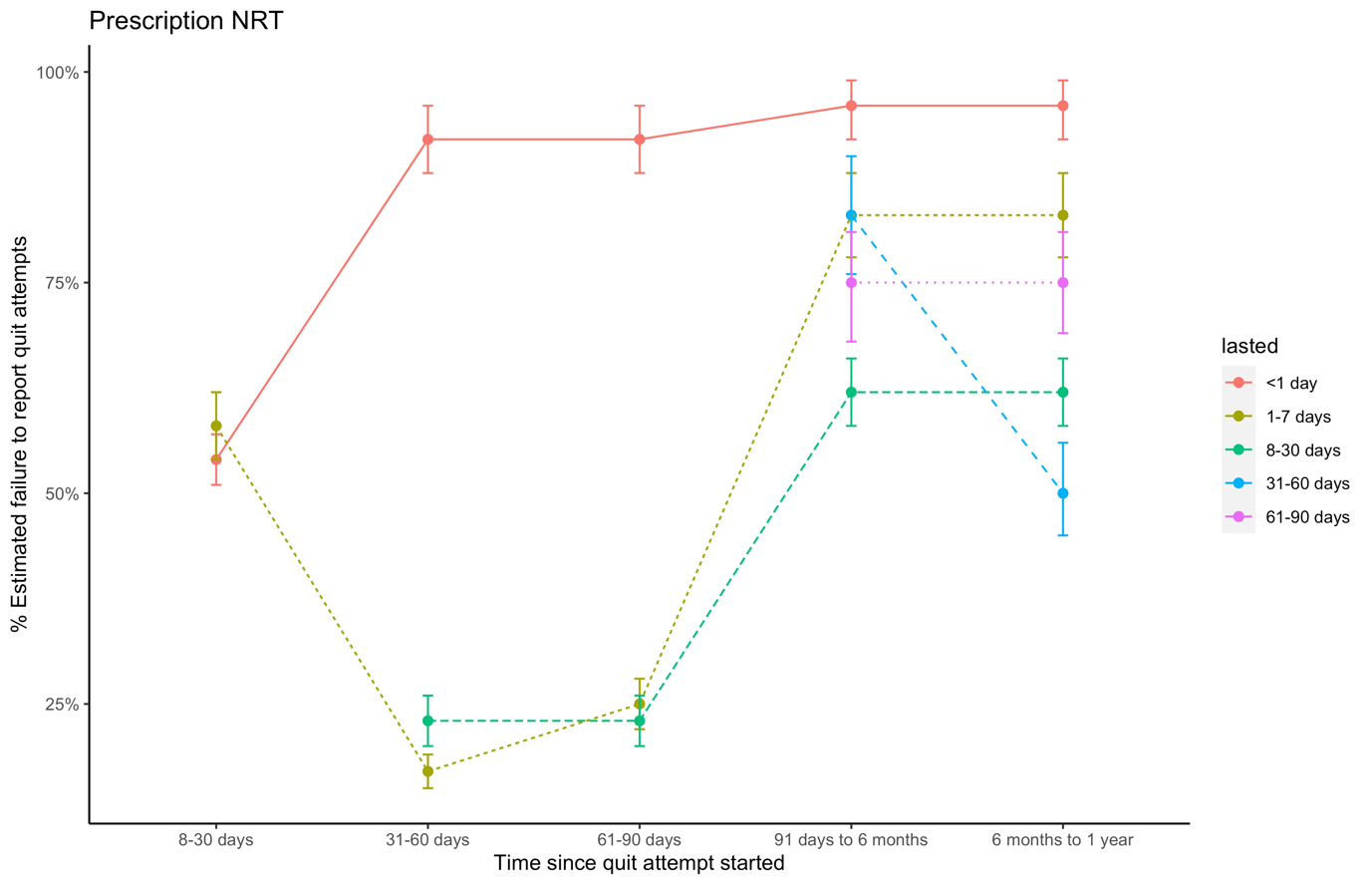
**

**
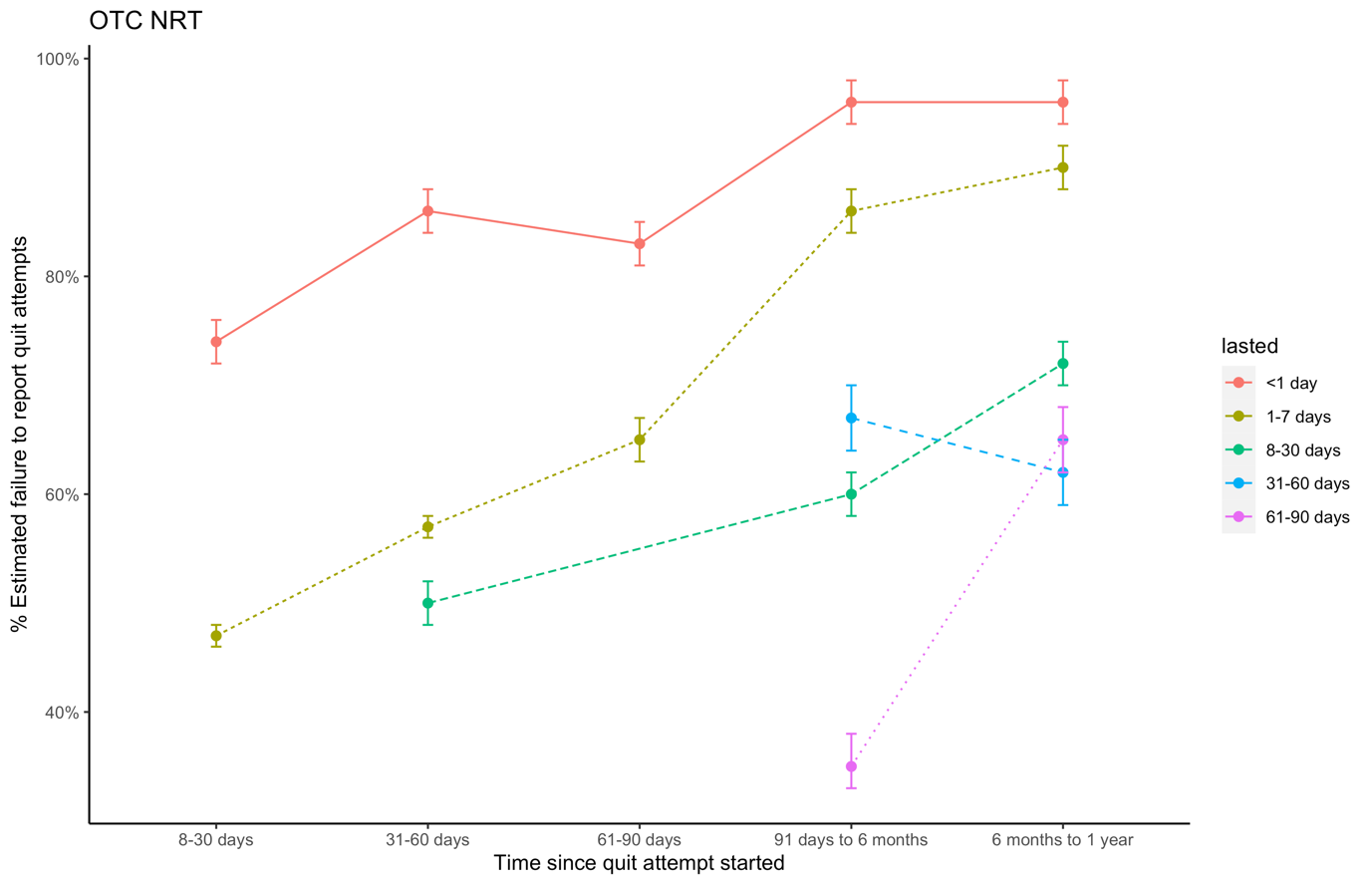
**

**
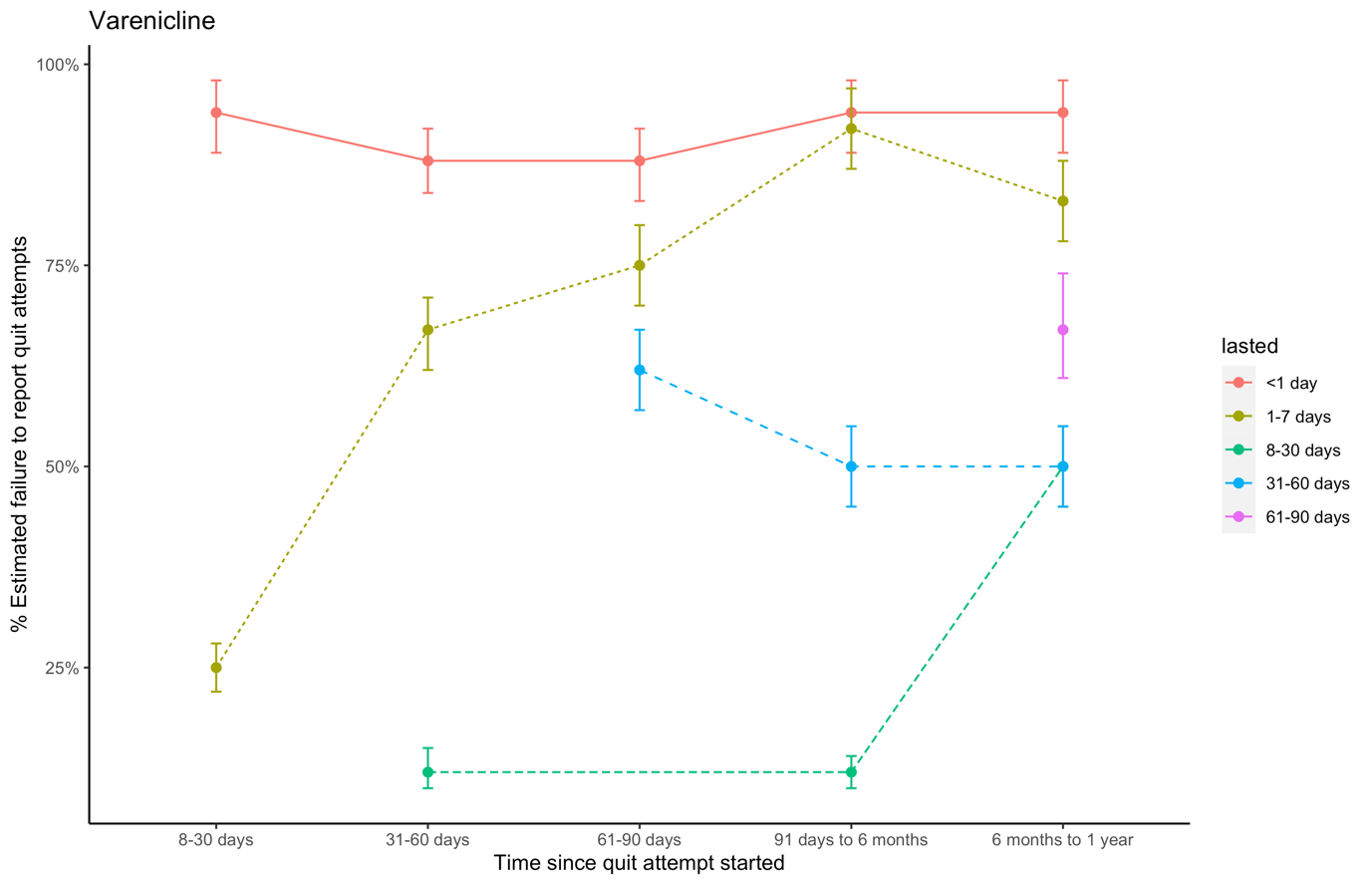
**


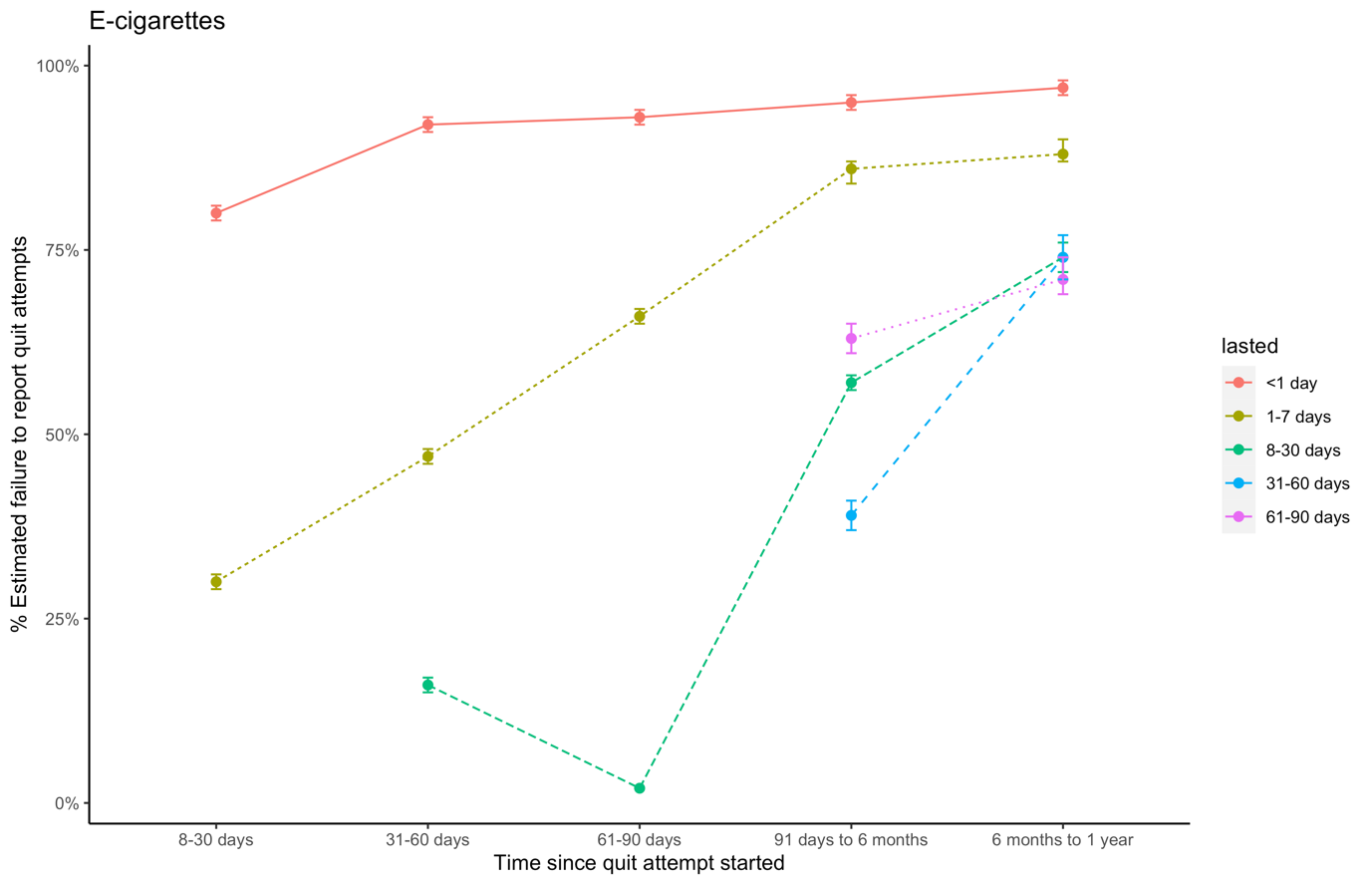


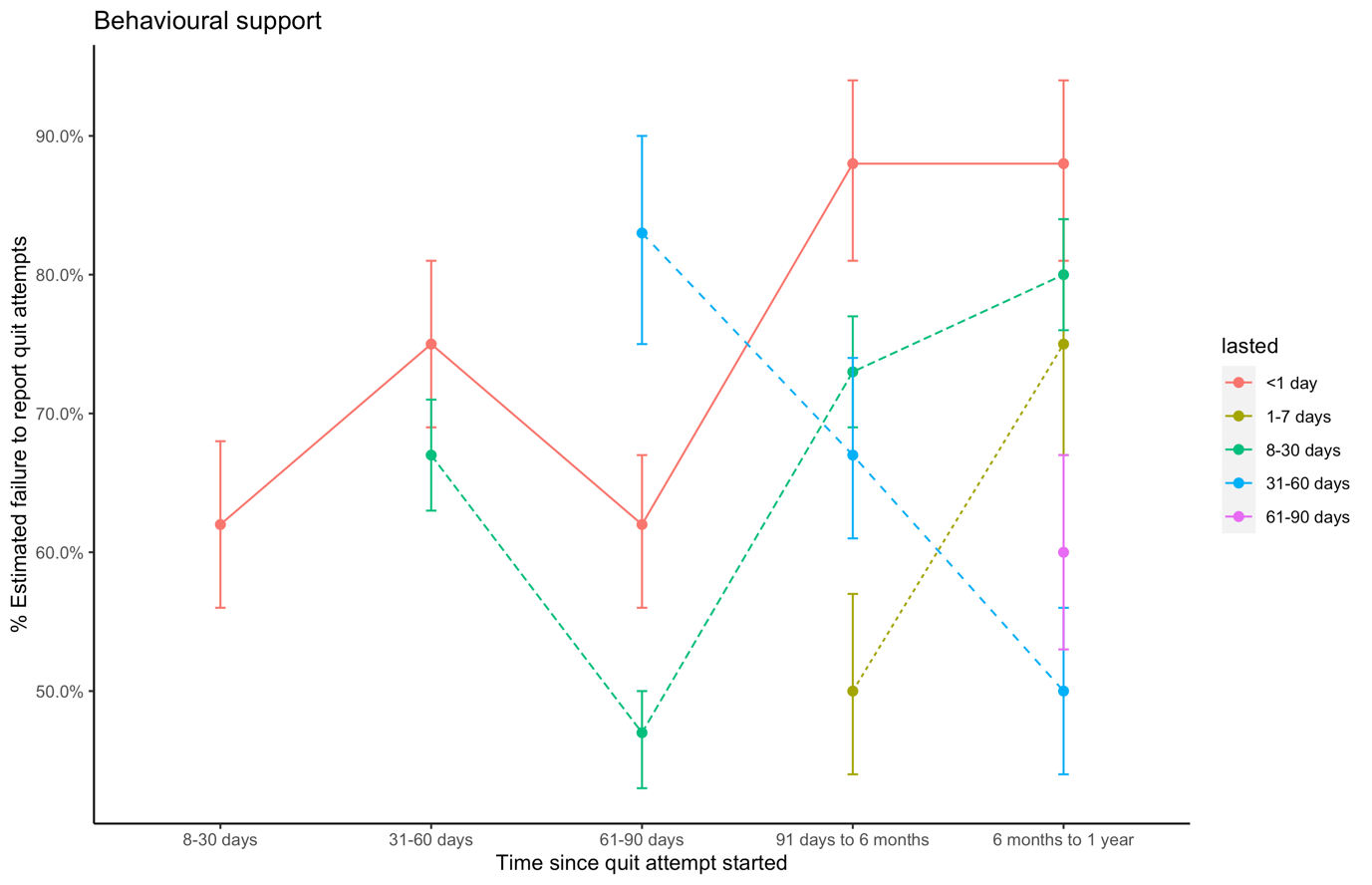


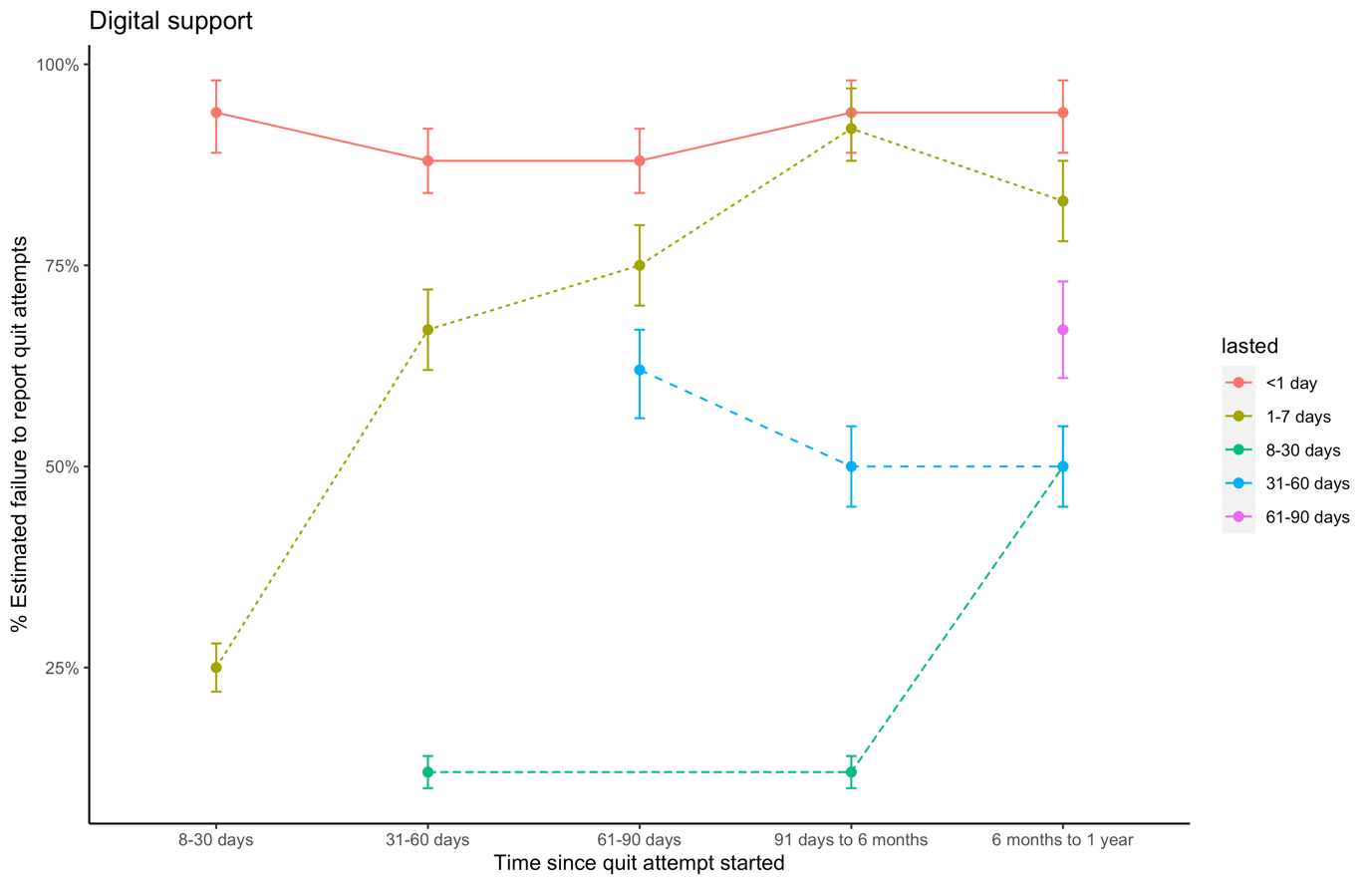


*Sensitivity analyses*


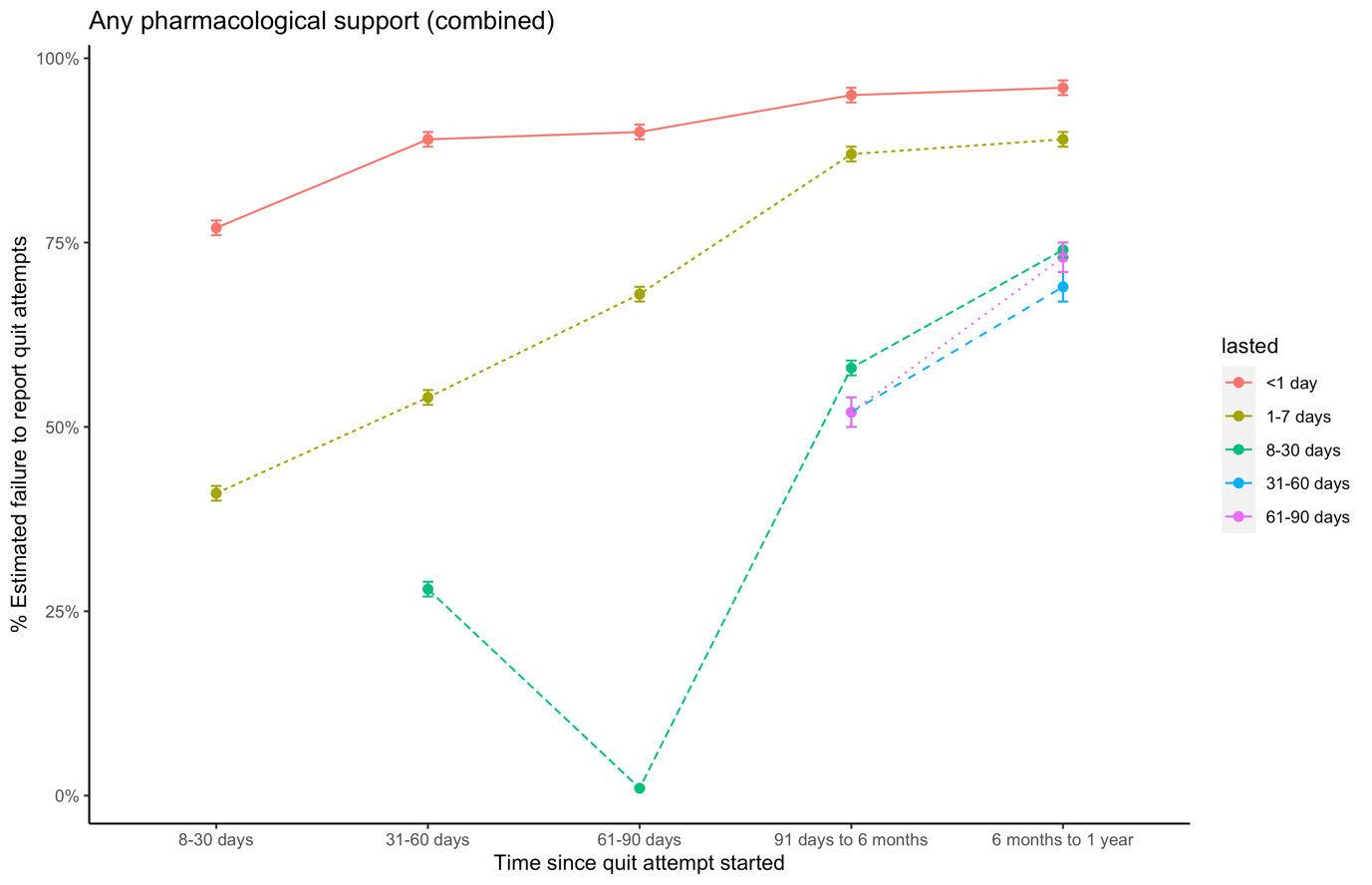


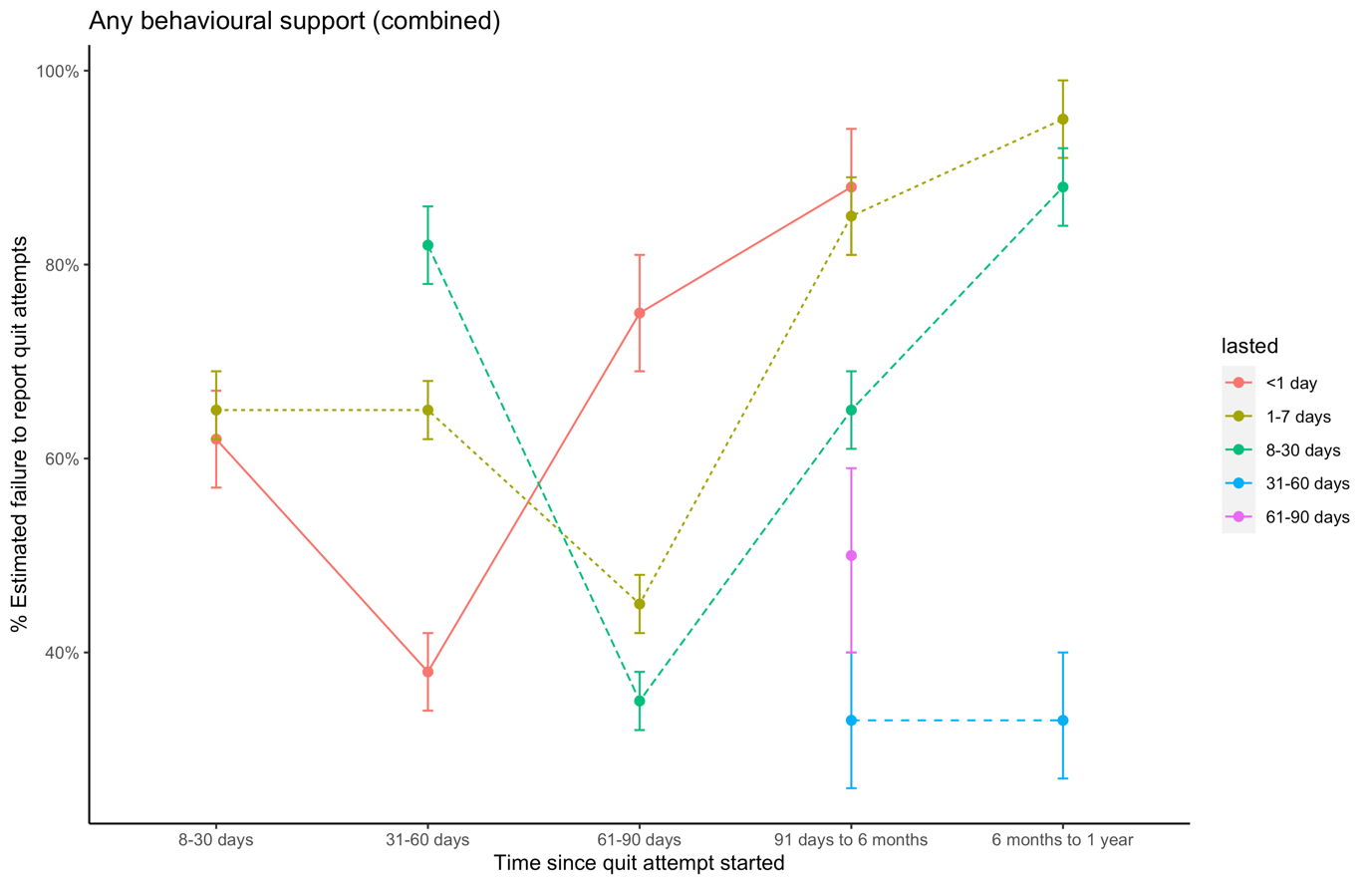


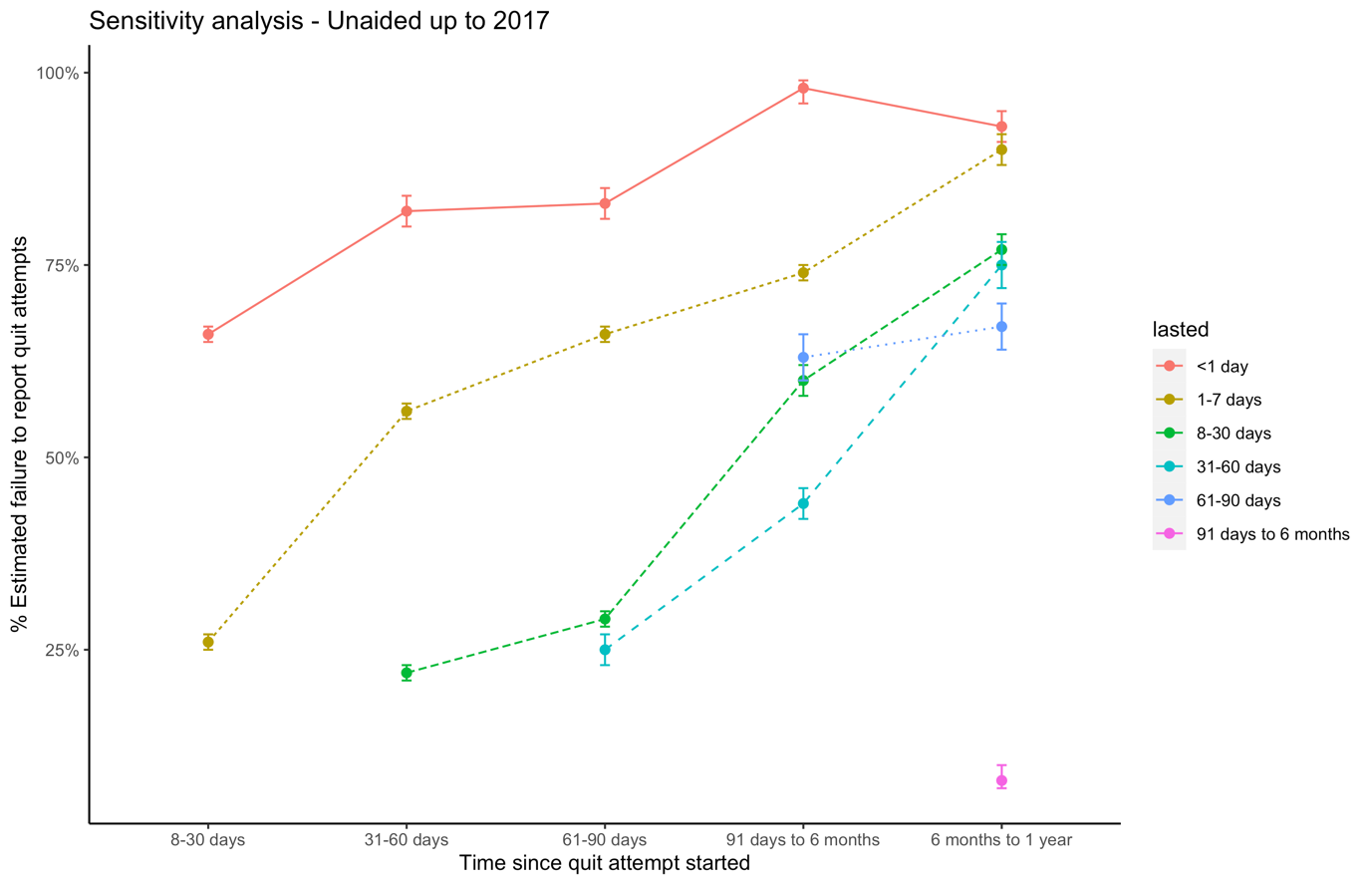

Supplement: Supplementary 2 — Supplementary File 2: percentage estimated failure to report quit attempts of varying lengths (indicated by the line colour) and varying times since the quit attempt started (x-axis) for each cessation aid, any pharmacological aid, any behavioural aid, or unaided attempts with data up to 2017 (sensitivity analyses). [file 5572480.f2.docx]
